# Species Delimitation and Phylogeography of *Aphonopelma hentzi* (Araneae, Mygalomorphae, Theraphosidae): Cryptic Diversity in North American Tarantulas

**DOI:** 10.1371/journal.pone.0026207

**Published:** 2011-10-12

**Authors:** Chris A. Hamilton, Daniel R. Formanowicz, Jason E. Bond

**Affiliations:** 1 Auburn University Museum of Natural History and Department of Biological Sciences, Auburn University, Auburn, Alabama, United States of America; 2 Department of Biology, The University of Texas at Arlington, Arlington, Texas, United States of America; Biodiversity Insitute of Ontario - University of Guelph, Canada

## Abstract

**Background:**

The primary objective of this study is to reconstruct the phylogeny of the *hentzi* species group and sister species in the North American tarantula genus, *Aphonopelma,* using a set of mitochondrial DNA markers that include the animal “barcoding gene”. An mtDNA genealogy is used to consider questions regarding species boundary delimitation and to evaluate timing of divergence to infer historical biogeographic events that played a role in shaping the present-day diversity and distribution. We aimed to identify potential refugial locations, directionality of range expansion, and test whether *A. hentzi* post-glacial expansion fit a predicted time frame.

**Methods and Findings:**

A Bayesian phylogenetic approach was used to analyze a 2051 base pair (bp) mtDNA data matrix comprising aligned fragments of the gene regions *CO1* (1165 bp) and *ND1-16S* (886 bp). Multiple species delimitation techniques (DNA tree-based methods, a “barcode gap” using percent of pairwise sequence divergence (uncorrected p-distances), and the GMYC method) consistently recognized a number of divergent and genealogically exclusive groups.

**Conclusions:**

The use of numerous species delimitation methods, in concert, provide an effective approach to dissecting species boundaries in this spider group; as well they seem to provide strong evidence for a number of nominal, previously undiscovered, and cryptic species. Our data also indicate that Pleistocene habitat fragmentation and subsequent range expansion events may have shaped contemporary phylogeographic patterns of *Aphonopelma* diversity in the southwestern United States, particularly for the *A. hentzi* species group. These findings indicate that future species delimitation approaches need to be analyzed in context of a number of factors, such as the sampling distribution, loci used, biogeographic history, breadth of morphological variation, ecological factors, and behavioral data, to make truly integrative decisions about what constitutes an evolutionary lineage recognized as a “species”.

## Introduction

*“The recognition of the reality of species is the key to explaining organic diversity”* [1]

The spider genus *Aphonopelma*, the only genus of tarantula known to North America, is distributed throughout the southern third of the United States, west of the Mississippi River to California and down into the Central American Neotropics. Despite the charismatic nature of the group (large bodied and “hairy”), the taxonomy of *Aphonopelma* has received comparatively little attention and has been largely reliant upon sparse and sometimes poorly defined morphological data. Historically, three major efforts [2]–[4] have been undertaken to evaluate the taxonomic status, morphological character variation, and relationships of *Aphonopelma*, none of which employed an explicit phylogenetic approach.

Unfortunately, much of the past descriptive work was based on only one or two specimens, and generally lacked consideration of the wide range of intraspecific variation noted within group. Morphology-based phylogenies of tarantulas and other related mygalomorph taxa seem to signal widespread problems in homoplasy among morphological characters [5]–[9]. Also, the quantitative or meristic features often used to evaluate relationships among these taxa may be problematic [10], [11]. Not surprisingly, many arachnologists have lamented the present state of theraphosid taxonomy due to the morphological characters employed for diagnostic use [4], [5], [12]–[16]. Raven [17] considered the family Theraphosidae a “nomenclatural and taxonomic nightmare”. Accurate delimitation of species boundaries is especially important in taxa where one or more morphological characters are uninformative or are in conflict. Hebert *et al* [18], [19] suggest traditional taxonomy in morphologically conserved groups can lead to incorrect identifications and may fail to recognize cryptic taxa. Because many of the described tarantula species in the United States can be difficult to differentiate based on morphological characteristics alone, molecular data may prove to be an additional source of characters to evaluate taxonomy, species delineation, and to uncover cryptic species in this charismatic yet understudied group.

The objective of the study presented herein is to reconstruct the phylogeny of the *hentzi* species group and sister species across *Aphonopelma* taxa distributed in the eastern portion of the southwest United States, using a set of mitochondrial DNA markers that include the animal “barcoding gene” [18], [19]. We then employ these data, through the testing of multiple species delimitation techniques – DNA tree-based methods, a “barcode gap” using percent of pairwise sequence divergence (uncorrected p-distances), and the GMYC method – to consider questions regarding species boundary delimitation. Subsequently, we evaluate timing of divergence events for these delimited species to infer historical biogeographic events that have played a potential role in shaping the present-day diversity and distribution. The use of tree-based methods for species delimitation recognizes “species” as historical lineages [20] and has been shown to be effective when used in conjunction with uncorrected p-distance data outlined in Hebert *et al* [18], [19] and in a number of other studies [20]–[27].

We have chosen to make use of mitochondrial genes, as we are particularly interested in addressing the efficacy of cytochrome *c* oxidase subunit 1 (*CO1*) and ribosomal large subunit (*16S*) as species identification tools in both known and unknown groups within the genus *Aphonopelma* and other theraphosid taxa [18], [19], [28]. Mitochondrial genes have long served as preferred markers for phylogeographic and species-level phylogenetic analyses because of the speed with which they evolve -- mutations may reflect real substantive changes in metabolic pathways (e.g. cell respiration), leading to hybrid breakdown [29], [30]. As is the case with many understudied groups, we presently lack sufficient nuclear markers in mygalomorphs that would allow us to address divergence, coalescence, or gene-tree/species-tree issues.

Surprisingly, very little information is known about the overall genetic diversity and population structuring of tarantulas throughout their range [13], [31], [32]. *Aphonopelma* individuals possess traits that differ markedly from araneomorph spiders and other arthropods. Related burrowing mygalomorphs (e.g., trapdoor spiders in the genus *Aptostichus*) are thought to have very limited dispersal ability, as evidenced by clustering of individuals within habitat patches and by extreme structuring of populations at a genetic level [24], [33]–[37]. Because theraphosids do not “balloon” from the maternal site (a common form of dispersal where spiderlings will release strands of silk and ride air currents away from their natal site) their limited dispersal abilities, long life spans (15–30 years), indeterminate growth, and delayed sexual maturity (4–7 years to reach maturity) make them especially vulnerable to factors such as habitat destruction and stochastic processes [12], [35], [38]–[40].

### *The* Aphonopelma hentzi *species group*

The first tarantula described from the United States in 1854 was *Aphonopelma hentzi* [41]. The type specimen is believed to have been collected in southwestern Oklahoma [42], with the *A. hentzi* range extending from southwestern Missouri, through southern Kansas, southeastern Colorado, northeastern New Mexico, encompassing all of Oklahoma, the northern half of Texas, western Arkansas, and northwestern Louisiana [4], [13]. Smith [4] coined the term “*Aphonopelma hentzi* complex” which included: *A. arnoldi* Smith, *A. baergi* Chamberlin, *A. clarki* Smith, *A. coloradanum* (Chamberlin), *A. echinum* (Chamberlin), *A. gurleyi* Smith, *A. hentzi* (Girard), *A. hollyi* Smith, *A. odelli* Smith, *A. waconum* (Chamberlin), and *A. wichitanum* (Chamberlin). Murray [13] conducted an unpublished study of the *hentzi* species complex using a combination of genetic and morphological data. Her study redefined the intraspecific variation within the group, revealing that *A. hentzi* appeared to be a single wide-ranging species with extensive morphological variation, while hypothesizing the species exhibits high ecological plasticity throughout its wide distribution due to the diverse habitats where it can be found.

**Figure 1:**
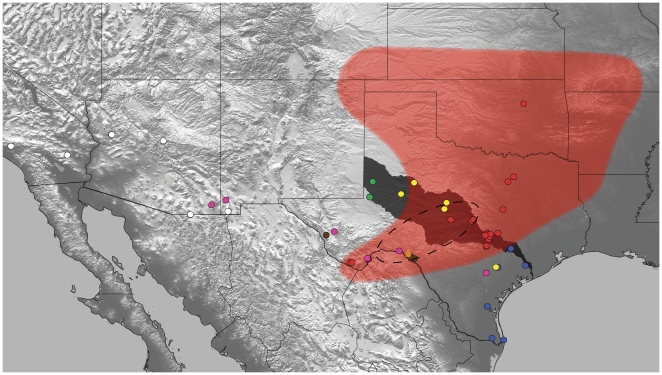
Sampled distribution map showing the 76 sampled *Aphonopelma* haplotypes. Colored circles represent sampling localities and correspond to species from the phylogenetic tree in Figures 1 & 2. The dark grey region represents the defined Colorado River Basin. Light red shading represents a generalized distribution map for *A. hentzi*. The dotted line circle corresponds to the ten independent PhyloMapper runs representing the area of hypothetical ancestral *hentzi* populations.

Previously, it was proposed that *A. hentzi* dispersed northwards after the Pleistocene's Last Glacial Maximum (LGM) as it tracked a desert-like ecosystem expansion into Missouri glade habitat (the northeastern-most area of its distribution) around 8,000–4,000 years ago [40]. We therefore predicted and tested the hypothesis that *A. hentzi* experienced a recent, rapid range expansion following the LGM, dominating new open northern niches after spreading from postglacial refugia in Texas' Colorado River Basin. Historically, the Colorado River cuts a path through the heart of Texas where today it appears to represent an area of species transition; a region north of which no other *Aphonopelma* species, except for *A. hentzi*, are thought to be found (Figure 1). The history of many lineages in the south-central United States (a region with 20 nominal tarantula species) has undoubtedly been complicated by repeated vicariance, extinction, and subsequent dispersal/expansion events. A comparative phylogeographic analysis of the North American *Aphonopelma* may lend insight into how the numerous glacial cycles of the Pleistocene affected the biodiversity of this region.

## Methods

### Taxon sampling

In an effort to sample the breadth of the *A. hentzi* species complex (defined above) distribution and phylogenetic diversity, we sampled 147 specimens from across Texas and the southwest United States from 15 sites visually identified as suitable habitats within the Colorado River Basin (hereafter referred to as CRB) in central Texas and 41 localities north, south, and west of the CRB. In reference to the *hentzi* complex, specimens were collected from areas near to and including five of the eleven type localities, as well as from across the breadth of the known distribution [13]. Outgroup individuals were collected from an assortment of western US material (California and Arizona), which in morphological work by Prentice [15] and in preliminary molecular data was shown to be sister to this material, yet highly divergent.

We attempted to collect ≥3 individuals at each site, per Wiens and Penkrot [20], though it was not always possible for localities where individuals were rare. We generally tried to collect adult females and mature males, but also included immature spiders if adults were not available in sufficient numbers (Table S1). Adult females are easily recognizable based on the morphology anterior to the epigastric furrow. Mature males are sexually dimorphic, possessing pedipalps modified as copulatory devices, (palpal bulbs). Juveniles were identified via morphology (when possible) or their sequences were “blindly” added to the analysis and later confirmed via simple association of DNA sequence similarity with *identified* adult specimens collected at or near the same locality. Each previously described species (*A. hentzi*, *A. moderatum*, *A. armada*, *A. anax*) was noted to be morphologically recognizable *ex invicem*. Specimens were collected from state lands with permission from Texas Parks & Wildlife or from private lands with permission from the landowner, preserved in 80% ethanol, and assigned a unique voucher number. At the conclusion of our studies, specimens will be deposited in the American Museum of Natural History, California Academy of Sciences, and Auburn University Museum of Natural History collections.

### Molecular Protocols

Tissue samples were taken from specimens using the technique of inducing limb autospasy of leg III on the right side of the spider [43]. In response to pressure, the limb will detach and the muscles will contract to prevent hemolymph loss. Genomic DNA was extracted using the Qiagen DNeasy Tissue Kit™ (Qiagen, Valencia, CA). The concentration of the extracted DNA was quantified by using spectrophotometry (NanoDrop ND-1000, Thermo Scientific, Wilmington, DE) or through gel electrophoresis. Legs were preserved in 100% ethanol or RNA*later*™ (Qiagen, Valencia, CA) and stored at −80°C. Muscle tissue was extracted from the leg by making a small incision on the ventral face of the femur and then cutting out ∼25mg of tissue.

DNA amplification was performed using the polymerase chain reaction (PCR) for the mtDNA “barcoding” gene regions *CO1* & *ND1-tRNA(Leu)-16S*. PCR and direct sequencing primers are listed in Table 1. Unincorporated dNTPs, primers, and other impurities were removed using ExoSAP-IT (USB Corporation; Cleveland, OH) prior to sequencing with an ABI 3130 Genetic Analyzer (Applied Bio-systems, Foster City, CA) using the ABI Big Dye Terminator version 3.2 Cycle Sequencing Ready Reaction Kit. Sequencing products were purified using Sephadex G-50 (Sigma-Aldrich, St. Louis, MO). All sequences were manually edited using the program Sequencher (ver. 4.1.2, Genecodes, Madison, WI). Mitochondrial DNA sequences were deposited in GenBank under the accession numbers JF803303 – JF803422 and JF907056 – JF907175 for *COI* and *ND1-16S* respectively (Table S1).

**Table 1:** PCR primers used to amplify and sequence the DNA barcoding regions *CO1* and *ND1-16S*.

PRIMER	PRIMER SEQUENCE	DIRECTION
***CO1***		
LCO1490	GGTCAACAAATCATAAAGATATTGG	Forward
LCO1490aphonopelma	TTTCTACTAATCACAAGGATATYGG	Forward
LCO1490cg	TTTCTACTAATCACAAGGATATTGG	Forward
LCO1490chalcodes	TTTCCACAAACCACAGGGATATCGG	Forward
LCO1490reversum	TTTCTACTAATCATAGGGATATTGG	Forward
C1-J-1751“SPID”	GAGCTCCTGATATAGCTTTTCC	Forward
C1-N-2776	GGATAATCAGAATATCGTCGAGG	Reverse
***ND1-16S***		
N1-J-12261	TCRTAAGAAATTATTTGA	Forward
LR-N-13398 (16Sar)	CGCCTGTTTAACAAAAACAT	Reverse
LR-N-12945	CGACCTCGATGTTGAATTAA	Internal reverse

### Alignment and Phylogenetic Analyses

Sequences were aligned with MUSCLE version 3.6 [44] using default parameters, followed by minor adjustments in MESQUITE version 2.73 [45]. The uncorrected pairwise genetic distances (p-distances) for these taxa were calculated in PAUP* 4.0b10 [46] then averaged for each defined species group in Excel (Microsoft Corporation, Santa Rosa, CA). The computer program Kakusan 3 [47] was used to determine the appropriate model of DNA substitution by Bayesian Information Criterion (BIC). Each gene region was partitioned by codon position; separate models were chosen for each.

Maximum Likelihood (ML) analyses were carried out in RAxML-7.2.8 [48] for the individual datasets, as well as the concatenated dataset, using the rapid-hill climbing mode. Parameters for the analyses incorporated the GTRGAMMA model of evolution based on 100 random addition sequence replicates; branch support values were computed via 100 non-parametric bootstrap replicates.

MrBayes ver. 3.1.2 [49]–[51] was used to infer the phylogeny using the models of DNA substitution indicated by BIC. The analysis of the concatenated dataset comprised eight Markov Chain Monte Carlo (MCMC) chains run for 15,000,000 generations. Trees were saved to file every 100 generations. Conservatively, topologies in the first 20% of the posterior distribution were discarded as burn-in following visual inspection in the program Tracer v1.5 [52]. The two independent runs were considered to have converged when the standard deviation of split frequencies value was <0.01. Clade posterior probabilities were computed from the remaining trees. The reported likelihood scores for all topologies post burn-in were computed using the “*sump*” command in MrBayes. Trees prior to burn-in and convergence were discarded using the “*sumt*” command. A 50% majority-rule consensus tree was produced. Trees were rooted by outgroup comparison using highly divergent sister taxa from the western United States.

### *Aphonopelma hentzi* haplotype network

To precisely estimate relationships among closely related haplotypes within the *A. hentzi* lineage, we employed statistical parsimony using the computer program TCS 1.21 [53] to create a haplotype network, with a 95% confidence level. Calculating how derived populations were on average, the number of mutational steps of each haplotype from the ancestral haplotypes was counted. The “ancestral” haplotype was identified by its internal position within the network, the number of lineages branching out from this position, and by the frequency of the haplotype within the analysis. Derived haplotypes are connected to the remainder of the network by one connecting branch [54]. Because our markers were mitochondrial and thus linked, we produced a single network using the concatenated dataset (which included missing data from 5 shortened *ND1-16S* sequences, 4 shortened *CO1* sequences, and ambiguities in 6 specimens).

### Delimitation of Species Boundaries

First, we applied a method that combines the approach described by Wiens and Penkrot [20] with a barcode gap metric. The Wiens and Penkrot method delineates species using molecular phylogenetics and evaluations of lineage isolation that assume no gene flow occurs or can occur between species. Originally, the method suggested integration with Templeton's Nested Clade Analysis [55], but NCA has been shown to suffer from some problems [56] particularly when applied to highly structured populations and species (a situation for which it was never intended). Instead, we used a combination of their tree-based method with a traditional DNA barcoding method, the “barcode gap”. Once a phylogeny had been inferred, species groups were identified based on clade exclusivity combined with our morphological knowledge of the previously identified species (*A. hentzi*, *A. moderatum*, *A. armada*, *A. anax*), and then a “barcode gap” was set at 6%, which was greater than the greatest intraspecific uncorrected p-distance (see Results).

Second, we employed the Pons *et al* [26] ‘generalized mixed Yule coalescent’ method for species delimitation (implemented in R (http://www.R-project.org/) using the ‘splits’ package (SPecies LImits by Threshold Statistics, http://r-forge.r-project.org/projects/splits/) [57] on a gene tree dataset of 76 non-identical haplotype taxa. This quantitative method provides a test of species boundaries by attempting to measure the degree of mtDNA genetic clustering by detecting a threshold value at the transition from interspecific to intraspecific branching patterns. The likelihood of the model, that separate coalescent clusters are nested within the tree, is compared to a null model for which the entire sample is derived from a single species. Nodes prior to the transition point are considered to be cladogenetic events that split species and subsequent nodes reflect coalescence within a species with the goal of delimiting independent evolutionary clusters. For the analysis, all identical haplotypes were first removed from the ML tree to render the tree fully dichotomous using the computer program TreeEdit [58] to resolve all zero branch lengths. The ML tree for our concatenated dataset was converted to ultrametric in R using the ‘*chronopl*’ command in Ape (Analyses of Phylogenetics and Evolution) [59], which implements the penalized likelihood (PL) method developed by Sanderson [60]. The ML tree was linearized with an arbitrary root set to 1, the default. Both the single threshold [26] and multiple threshold [61] models were performed on the dataset. The modified multiple threshold model allows for the speciation-coalescent transition to vary across the phylogenetic tree. The GMYC model calculates, as part of its output, a confidence interval (CI) that corresponds to the number of clusters within 2log*L* units of the ML estimate.

### Tests of Neutrality & Population Expansion

The demographic histories for the *A. hentzi* clade and the “northern” *hentzi* group (defined here as all specimens north of the Colorado River) were tested using Tajima's D [62], [63] and Fu's F_ST_ [64], [65], where negative values indicate population expansion or historical bottleneck [66]. Both statistics and their significance were assessed using 10,000 samples simulated under a model of constant population size in Arlequin 3.5.1.2 [67]. This method has been previously used to track post-Pleistocene population expansions from refugia [68]–[71].

### Estimating divergence times

No study to date has attempted to estimate divergence dates or rates of mtDNA evolution within the Theraphosidae. Obtaining estimates of divergence are generally based upon known historical events, geologic or fossil, which can be used as calibration points for a taxon-specific mutation rate estimate (determined from local molecular clock rates). For this study no such fossils are available, so we were forced to confront this problem using a two-pronged approach in hopes of converging on the most plausible mtDNA rate of evolution in this group of spiders. In order to tackle this problem, we use a mygalomorph substitution rate estimate of 4% [34] to evaluate the correspondence with dating events that likely occurred during the Pleistocene. Alternatively, we estimate substitution rates using historical biogeographic events for calibration of cladogenesis among and between species across the tree. The later approach serves only as an internal check as it represents certain circularity with respect to the hypotheses we wish to evaluate, which are related to how Pleistocene glaciation events shaped the present day distribution of the *Aphonopelma* genus.

Past studies of arthropod divergence times have traditionally used the 2.3% standard insect clock [72], however we employed a 4% pairwise divergence per million years (a rate estimated from *16S*) determined from a study of a related mygalomorph genus, *Aptostichus* [34]. Node ages for species and population divergence were estimated from the concatenated data set using the computer program BEAST v1.6.1 [73]. For population divergence estimation within *A. hentzi*, specimens were assigned to one of four biogeographic-based groups within the larger species group: one group comprising all *hentzi* representatives (denoted as *h* in Table 2), a group of all the specimens within the CRB and everything to the north (*h*CR), a group representing all specimens north of the Colorado River (*h*CRN), and a northern group representing every specimen north of the CRB (but not including the CRB) (*h*N). With the exception of the *hentzi* clade, monophyly was not enforced on these biogeographic groups in the BEAST analysis.

**Table 2:** BEAST divergence date estimation for two hypothetical mtDNA rates of evolution.

	Fixed Clock dates @ 4.0%	Calibration dates (used to estimate rate)	Yule birth-death Estimated dates @ 14.2%
*h*	925,000 ybp (95% CI = 1,099,800−753,800 ybp)	1,800,000 ybp +/− 0.1 (95% = 1.964−1.636 million ybp)	1,710,400 ybp +/− 1,378 (95% CI = 1.913−1.512 million ybp)
*h*CR	237,000 ybp (95% CI = 294,900−183,200 ybp)	500,000 ybp +/− 0.180 (95% = 796,000−204,000 ybp)	16,230 ybp +/− 1,335 (95% CI = 22,042−8,840 ybp)
*h*CRN	237,000 ybp (95% CI = 294,900−183,200 ybp)	18,000 ybp +/− 0.003 (95% = 22,900−13,000 ybp)	16,230 ybp +/− 1,335 (95% CI = 22,042−8, ybp)
*h*N	237,000 ybp (95% CI = 294,900−183,200 ybp)	6,000 ybp +/− 0.00125 (95% = 8,000−3,900 ybp)	8,640 ybp +/− 260 (95% CI = 10,789−6,477 ybp)

Groups are denoted as: *h*  =  the *hentzi* species clade; *h*CR  =  all specimens within the CRB and everything to the north; *h*CRN  =  all specimens north of the Colorado River; *h*N  =  every specimen north of the CRB (but not including the CRB).

The BEAST run for the fixed clock analysis comprised 120,000,000 generations, sampled every 1000 generations, using a 4% fixed molecular clock, assuming a Yule speciation model for the tree prior, with unlinked substitution models (using the same models chosen for the Bayesian analysis – see Results), and default options for all other prior and operator settings. In order to ensure convergence and correct effective sample size (ESS), two chains were run (one for 80,000,000 and one for 40,000,000) then combined using LogCombiner v1.6.1. Convergence was assessed by inspection of the trace plots and the effective sample sizes using Tracer v1.5. Burn-in was set to remove 20% of the total trees sampled. TreeAnnotator v1.6.1 was used to sum the trees, after burn-in, for the 50% majority-rule consensus ‘maximum clade credibility tree’ with ‘mean heights’ for the node heights.

For comparison, we estimated the mean rate of evolution across the tree by calibrating nodes/groups using historical biogeographic events (but see comments above). BEAST was run for 100,000,000 generations, sampled every 5000 generations, assuming a Yule birth-death speciation model for the tree prior from a randomly generated starting tree, with unlinked substitution models (using the same models chosen for the Bayesian analysis – see Results), and a normal distribution prior for all *tmrca* (calibration) nodes. To ensure convergence and correct ESS, 2 chains were run (50,000,000 each) and then combined. Convergence, burn-in, and the summing of trees were carried out the same as above.

The *hentzi* nodes that were calibrated (*h*, *h*N, *h*CRN, *h*CR) were set at dates presumed to be important not only to *hentzi* populations but to other similar splits seen across the tree: *h*  = 1.8 million ybp +/− 0.1 (95% interval of 1.964−1.636) set for the start of the Pleistocene; *h*CR  = 0.500 million ybp +/− 0.180 (95% interval of 0.796−0.204) set for the middle Pleistocene glacial extent into Kansas [74], [75]; *h*CRN  = 0.018 million ybp +/− 0.003 (95% interval of 0.0229−0.013) set for the LGM; *h*N  = 0.006 million ybp +/− 0.00125 (95% interval of 0.0080−0.0039) set for the 8,000−4,000 ybp spread into Missouri as proposed by Janowski-Bell [40] (Table 2).

### Ancestral Population Reconstruction

To assess the geographic locations of ancestral *Aphonopelma* nodes (hypothetically representing the MRCA) and potential historical paths of gene flow, we employed the likelihood method implemented in PhyloMapper1b1 [76] to test *a priori* hypotheses regarding Pleistocene refugia in Texas. PhyloMapper takes a known phylogeny with branch lengths and the geographic localities of all individuals represented at the tips of tree, using a spatially-explicit random walk model of migration, to calculate the likelihood of observing the haplotypes sampled at their current geographic locations, given the geographic coordinates of clade ancestors and the dispersal distance of the species [76].

PhyloMapper analyses were carried out on the concatenated dataset using the default settings of 10 replicates of smoothing and 100 replicates for optimization after setting the focal clade. Both the rate smoothing and optimization parameters require replicates be run in the analysis because the nonparametric rate smoothing procedure uses a hill-climbing approach and entrapment in a local optimum is possible [76]. We randomized the PhyloMapper approach by replicating the analysis 10 times in order to independently arrive at an average historical distribution and likelihood score for the *A. hentzi* ancestral population.

## Results

### Summary of Sequence Data and Genetic Variation

**Figure 2:**
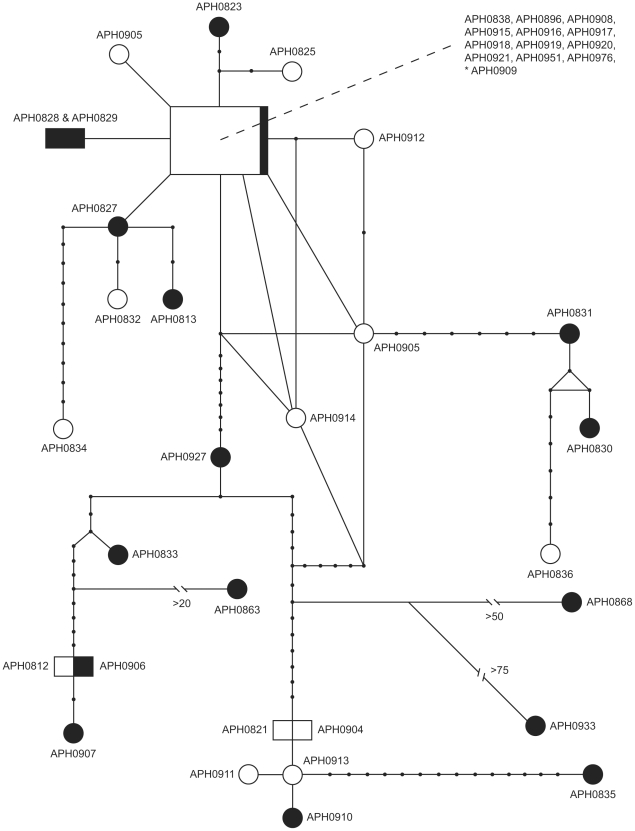
*Aphonopelma hentzi* haplotype network. Specimens sampled from across the *A. hentzi* distribution are represented in a haplotype network. Black objects represent haplotypes designated as “southern” haplotypes (south of the Colorado River) and white objects represent “northern” haplotypes (north of the Colorado River). The * denotes the APH0909 haplotype, the only “southern” haplotype represented in the most commonly shared haplotype box (proportionally separated between “northern” and “southern” haplotypes).

We sampled sequence data for 120 individuals comprising 2051 base pairs (bp) of a concatenated data matrix consisting of aligned fragments of the mtDNA gene regions *CO1* (1165 bp) and *ND1-16S* (886 bp). Primer fidelity across taxa was not always consistent in *CO1*, consequently some specimens have slightly truncated sequence lengths. Base frequencies for the combined dataset: A = 0.26417, C = 0.13909, G = 0.19785, T = 0.39890; uncorrected base pair frequencies appear homogenous (X^2^ = 52.229166, df = 357, p = 1.0). The data set contains 687 variable sites, 592 of which are classified as parsimony-informative characters. The complete data set contains 76 unique haplotypes, 27 of these sampled from the *A. hentzi* clade (Table S1; Figures 1 & 2). A statistical parsimony network shows 10 haplotypes represent the “northern” group, 13 in the “southern” group, with 4 being shared (2 within their group and 2 between groups). The most common haplotype is shared across 13 specimens (12 “northern” and 1 “southern”) (denoted by large box; Figure 2). Minor reticulation connects a small group of specimens, potentially due to missing data and ambiguities. The total nucleotide diversity, π, in the *A. hentzi* clade is 0.009552 (+/− 0.004809) (Table 3).

**Table 3:** Demographic history and genetic diversity in *Aphonopelma hentzi*.

	Tajima's D	Fu's Fs	Nucleotide diversity (π)	Specimens	Haplotypes	Haplotype diversity (Hd)
*hentzi*	−2.03906 (p = 0.00330)	−2.12372 (p = 0.26460)	0.009552 +/− 0.004809	42	27	0.9048 +/− 0.0409
*hentzi* (S)	−1.36558 (p = 0.07600)	1.61579 (p = 0.76270)	0.017496 +/− 0.009126	14	13	0.9670 +/− 0.0366
*hentzi* (N)	−2.13833 (p = 0.00360)	−3.98188 (p = 0.05230)	0.002622 +/− 0.001470	28	10	0.7910 +/− 0.0815

### Phylogenetic Analyses & “barcode gap” Species Delimitation

**Figure 3:**
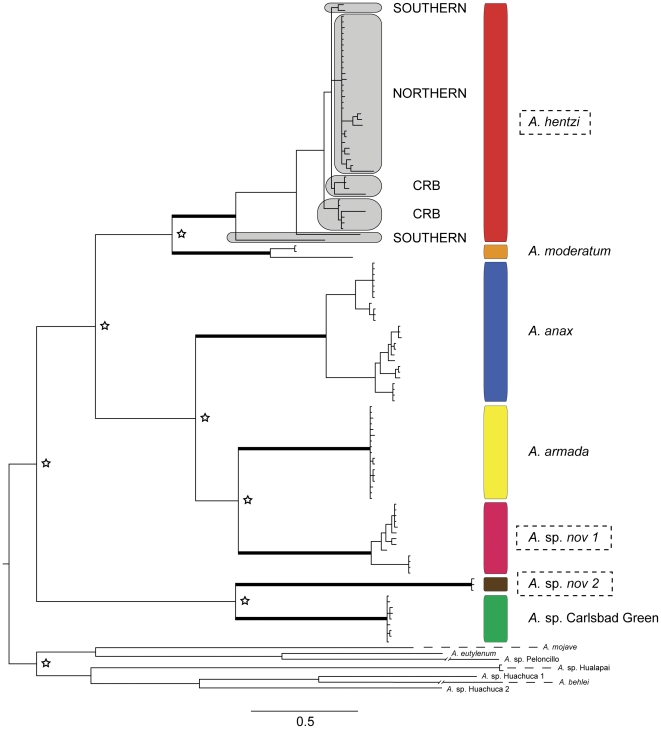
Inferred Bayesian mtDNA phylogeny for the *Aphonopelma* of Texas. The key (inset) references the seven species delimited in this analysis by species name (Red = *A. hentzi*; Orange = *A. moderatum*; Blue  =  *A. anax*; Yellow  =  *A. armada*; Pink  =  *A.* sp. *nov 1*; Brown  =  *A.* sp. *nov 2*; Green  =  *A.* sp. Carlsbad Green). These colors and species combinations are consistent throughout the figures. *A. hentzi* population designations are highlighted by light grey boxes. Outlined boxes around species names denote members of the *hentzi* cryptic species group. A thickened branch denotes species clades with Bayesian posterior probabilities of 1.00 and ML bootstrap values ≥95%. Support for major nodes with Bayesian posterior probabilities of 1.00 and ML bootstrap values ≥95% are denoted by the star symbol. Western United States species comprise the outgroup.

Based on the analysis in Kakusan, the following models of DNA substitution were used: *CO1*: codon 1 = HKY85 + Gamma, codon 2 = GTR + Gamma, codon 3; ND1: HKY85 + Gamma for all codon positions; *tRNA-Leu*: K80 + Gamma; *16S*: GTR + Gamma. Three independent Bayesian analyses, as well as Maximum Likelihood (ML), were run on this unlinked dataset: the *ND1-16S* region individually, the *CO1* region individually, and the concatenated matrix. In each independent analysis, as well as the concatenated dataset, each species clade was recovered as a strongly supported monophyletic group (posterior probability = 1.00; ML bootstrap ≥95%) (Figure 3). The ML analysis conducted in RAxML on the concatenated data set recovered a tree with a –log likelihood value of 12530.41. Along with the highly supported relationships, the topologies of all three analyses were indistinguishable.

All putative species clades are separated by >6% interspecific pairwise divergence (the “barcode gap”) (Table 4). Only two species pairs were under 7% pairwise divergence; *A. hentzi* – *A. moderatum* (6.84%) and *A. armada* – *A.* sp. *nov 1* (6.82%). Both of these species pairs consist of species that are clearly morphologically identifiable from each other. The average intraspecific divergence was <3% for all putative species clades. The highest intraspecific divergence was seen in the *hentzi* clade where the most southern specimen (and most basal in the clade), APH0933 from the Big Bend National Park area, was ≤5.7% divergence from the other *hentzi* specimens. Strikingly, there is only 0.16% divergence found within the “northern” population of *A. hentzi* (all specimens north of the Colorado River), while there is 2.6% in the “southern” group (all specimens south of the Colorado River).

**Table 4:** Average interspecific uncorrected pairwise genetic distances (p-distances) for the *Aphonopelma* found in Texas.

	*A. hentzi*	*A. moderatum*	*A. anax*	*A. armada*	*A.* sp. *nov 1*	*A.* sp. *nov 2*	*A.* sp. *Carlsbad Green*
*A. hentzi*	0	-	-	-	-	-	-
*A. moderatum*	6.84	0	-	-	-	-	-
*A. anax*	9.15	9.06	0	-	-	-	-
*A. armada*	9.26	8.58	7.61	0	-	-	-
*A.* sp. *nov 1*	**9.84**	9.61	7.91	6.82	0	-	-
*A.* sp. *nov 2*	**11.94**	12.05	12.65	11.36	**12.25**	0	-
*A.* sp. *Carlsbad Green*	11.44	11.14	11.91	11.11	11.84	9	0

**Figure 4:**
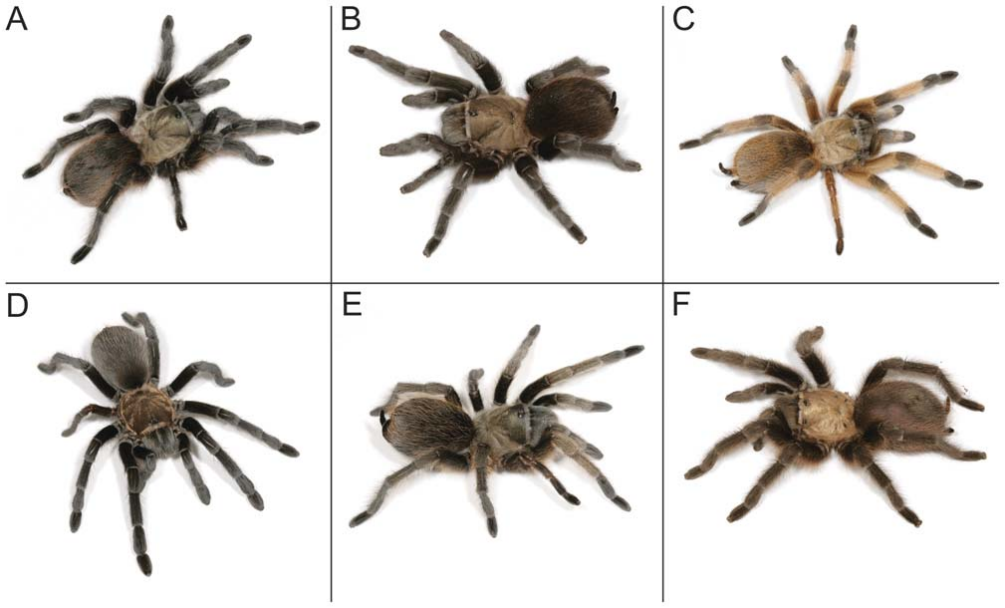
*Aphonopelma* species represented in this analysis. Sampled species consist of A – *A. hentzi*; B – *A. anax*; C – *A. moderatum*; D – *A. armada*; E – *A.* sp. Carlsbad Green; F. *A.* sp. *nov 1*. *A.* sp. *nov 2* is not pictured.

Based on the highly supported monophyly of these species clades, combined with the pattern and amount of genetic divergence seen in the “barcode gap” data, we postulate there may be as many as seven species in the eastern portion of the southwest United States (New Mexico east through Texas, Oklahoma, eastern Colorado, Kansas, Missouri, Arkansas, and Louisiana) (Figure 1). This group consists of *A. hentzi*, *A. anax*, *A. armada*, *A. moderatum* and three potentially undescribed species (*A.* sp. *nov 1*, *A.* sp. *nov 2*, and *A.* sp. Carlsbad Green – we are unsure at this time whether the latter is previously described but unrecognizable or is a new species) (Figures 3 & 4).

### Generalized Mixed Yule Coalescent species delimitation

**Figure 5:**
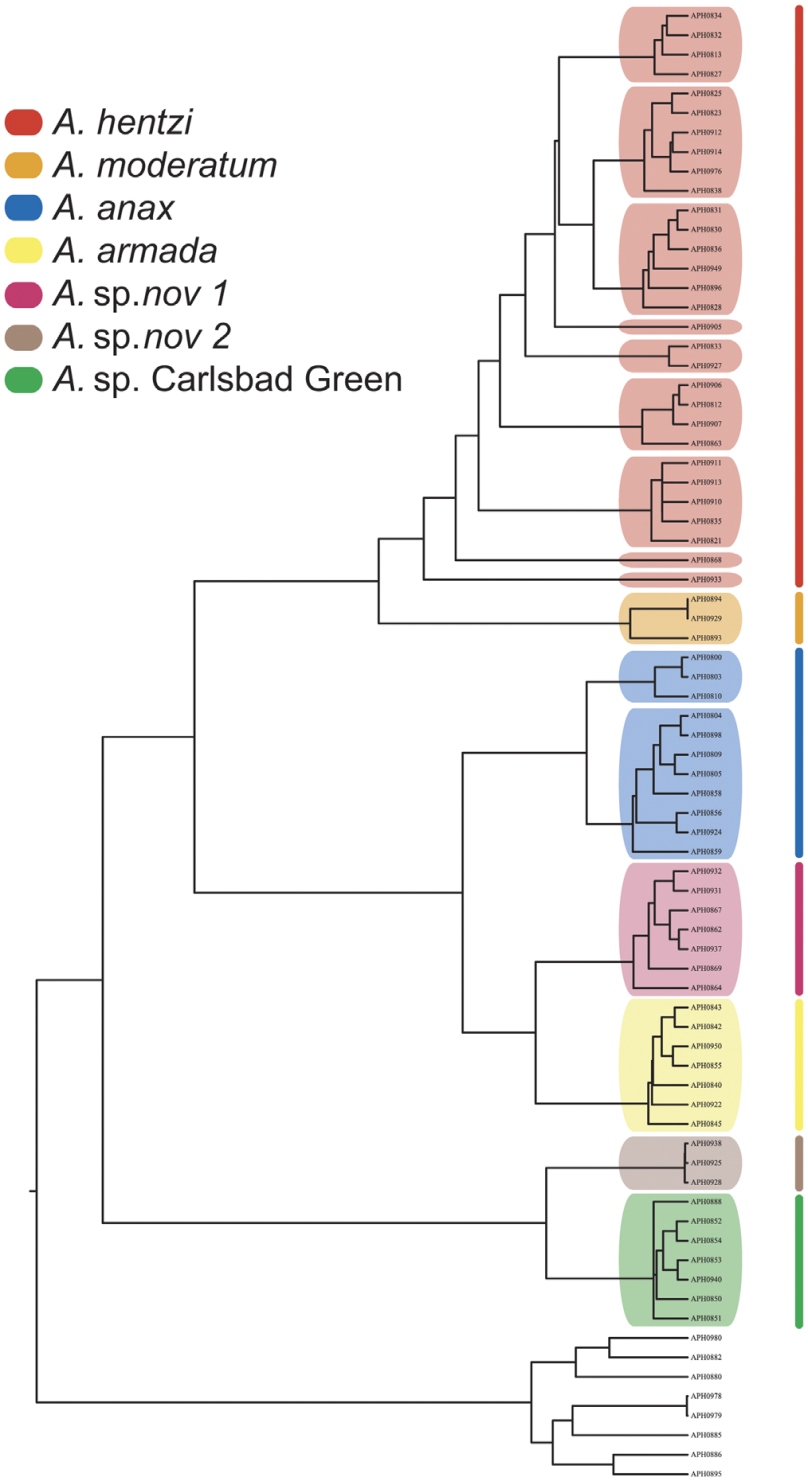
Generalized Mixed Yule Coalescent species delimitation tree. GMYC clusters delimited from the single-threshold model are highlighted and grouped according to their defined species. A total of 13 independent coalescence groups plus 3 singletons (singletons constitute a cluster given more population representatives) were delimited for the ingroup (14 total ML clusters; CI = 14–16). The data revealed strong geographic structuring and phylogenetic gene tree clustering in all putative species groups except *A. hentzi*. Species and color combinations correspond to those represented in Figure 1.

A comparison of the performance between the single and multiple threshold models revealed that the two methods were not significantly different from each other (X^2^ = 1.87539, df = 9, p = 0.9932862). Consequently, the results of the single threshold model are those we discuss. The GMYC model provided a significantly better fit to the data than the null model's hypothesis of the entire sample being derived from a single species with uniform branching (p = 0.0189) and indicated a total of 16 independent coalescence groups within the focal taxa (13 clusters plus 3 singletons - singletons constitute a cluster given more population representatives). The total GMYC analysis, including the outgroup, is represented by 14 ML clusters (CI = 14–16) and 23 ML entities (CI = 21–27) (Figure 5).

Delimited GMYC clusters (*A. armada*, *A.* sp. *nov 1*, *A. moderatum*, *A.* sp. *nov 2*, and *A.* sp. Carlsbad Green) were largely congruent with species clades defined by the tree-based methods and the “barcode gap” (Figures 3 & 5; Table 4). Exceptions included the *A. anax* species clade that was split into two clusters, corresponding to a previously observed phylogenetic split between the northern (those populations at or above the Colorado River) and southern populations (populations at or below the Colorado River), as well as the *A. hentzi* species clade splitting into nine clusters and singletons.

### Demographic History

The analysis of the tree topology for the “northern” group of *A. hentzi* indicates a lack of significant population structuring (Figure 3). We explored the demographic history of *A. hentzi* further by analyzing Tajima's D and Fu's F_ST_ to evaluate if this species experienced rapid population expansion and subsequently dispersed north in the recent past. The data revealed significant negative values for Tajima's D in the *A. hentzi* species clade (D =  −2.03906; p = 0.00330) and the “northern” group of *A. hentzi* (D =  −2.13833; p = 0.00360), indicating a rapid expansion of mtDNA haplotypes (Table 3). Ramos-Onsins and Rozas [66] showed Fu's F_ST_ to be a very powerful test in detecting past range expansion, especially for large sample sizes. The *A. hentzi* species clade exhibits a Fu's F_ST_ of −2.12372 (p = 0.26460), while the northern group of *A. hentzi* was −3.98188 (p = 0.05230) (Table 3). These results are significant for the northern group, though by a small margin with the p-value of 0.05. An increase of a wider range of sampling from more of the northern populations than is reported here may continue the trend and increase significance.

### Divergence Dating

Our dual approach was taken due to the unavailability of fossil date calibrations. This allowed us to test hypotheses pertaining to whether the Pleistocene played a major role in lineage splitting events for this group of spider. Analyses were carried out first by fixing a molecular clock and attempting to match cladogenetic events to geologic dates. We then calibrated historical biogeographic dates onto nodes and groups of specimens, allowing us to test whether these events and the associated estimated mtDNA substitution rate matched our preferred mygalomorph rate hypothesis.

**Figure 6:**
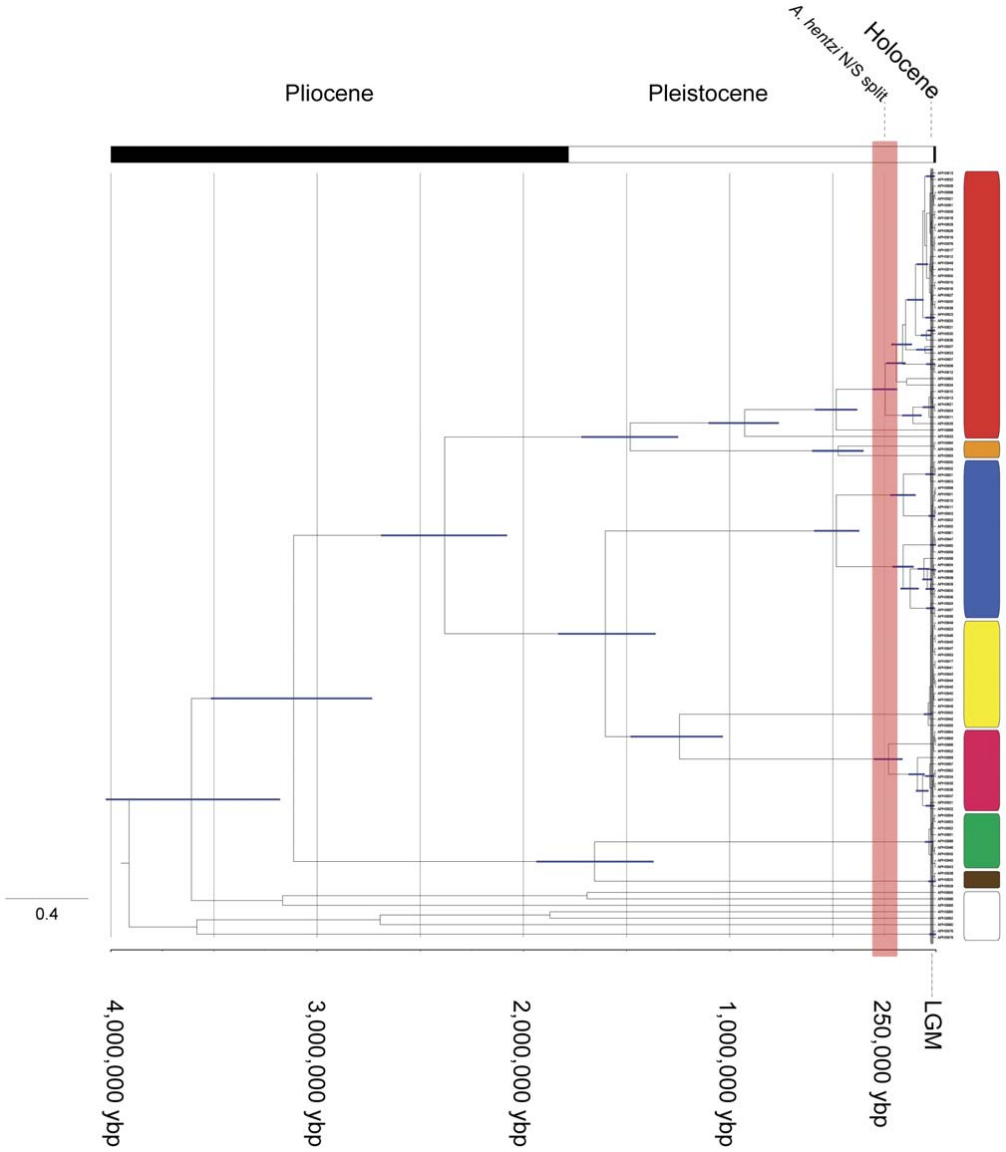
4% clock-constrained gene tree for the species of *Aphonopelma* found in Texas. To assign dates to population-splitting events within the *A. hentzi* lineage and assess the importance of these historical events on cladogenesis across the whole tree, a fixed molecular clock at 4% was applied in BEAST to evaluate how this mygalomorph rate of evolution relates to the events of the Pleistocene. Population splits within the *A. hentzi* clade (northern vs. southern) are predicted to have originated around 237,000 ybp (294,900−183,200), represented by the light red box. The Pliocene/Pleistocene/Holocene time frame is displayed at top, years from present on the bottom, with The Last Glacial Maximum designated by the grey box. The bars correspond to 95% confidence intervals.

Although a number of studies [37], [77]–[82] used the general arthropod molecular clock of 2.3% pairwise divergence per million years for mitochondrial genes in insects and arachnids, first employed by Brower [72], to test our hypotheses we preferred to use a rate previously derived from the same infraorder of spiders, the trapdoor genus *Aptostichus* [34]; also used recently by Cooper *et al* [83]. Fixed clock (4%) divergence dates for population fragmentation and subsequent expansion of the “northern” designated *A. hentzi* (*h*N, *h*CRN, *h*CR) were estimated at 237,000 ybp (95% CI = 294,900−183,200 ybp). The split of all extant *A. hentzi* populations was determined to correspond to 925,000 ybp (95% CI = 1,099,800−753,800 ybp) and around 1.75 to 1.25 million ybp between the *A. hentzi* lineage and the *A. moderatum* lineage (Table 2; Figure 6).

Based on node calibrations using historical biogeographic events hypothesized to have played a role in lineage divergence we obtained an unusually high substitution rate estimate of 14.2% per MY. These node calibrations and resultant rate estimate places the split of all extant *A. hentzi* (*h*) populations at 1,710,400 million ybp (95% CI = 1.913−1.512 million ybp), the *h*CR and *h*CRN populations at 16,230 ybp (95% CI = 22,042−8,840 ybp), and the *h*N populations at 8,640 ybp (95% CI = 10,789−6,477 ybp) (Table 2), with the *A. hentzi* lineage and the *A. moderatum* lineage splitting around 2.5 to 1.5 million ybp (Figure 6).

The dates inferred from the 4% *Aphonopelma* mtDNA reflect population fragmentation and species divergence occurring during the Pleistocene and back into the Pliocene, supporting the hypothesis that the events within this time period played a significant roll in the population fragmentation across *Aphonopelma*. With the 4% rate seemingly more logical and consistent with other similar studies [83], going forward we will discuss the results associated with this mygalomorph rate of substitution. Additional cladogenetic events are worth noting and appear to be timed with the same historical events that affected *A. hentzi*. At the same time that the southern *A. hentzi* haplotypes were fragmented, ∼500,000 ybp (4%), two other species exhibit significant population splits; the *A. moderatum* lineage possesses a deep fragmentation of the western and eastern populations, and a northern group of *A. anax* (also found within the CRB) was isolated from its sister clade of southern haplotypes, building up considerable mtDNA divergence (Figures 3 & 6). During the same relative time period that *A. hentzi* and *A. moderatum* split (∼1.6−1.5 million ybp (4%)), two other important branching events occur across the tree; the *A. anax* lineage split from the *A.* sp. *nov 1*/*A. armada* ancestor, and the *A.* sp. Carlsbad Green and *A.* sp. *nov 2* lineages split (Figure 6).

### Ancestral Population Reconstruction

Attempting to reconstruct ancestral population localities gives us a quantifiable approach from which to assess the likelihood that the ancestral node of definable *A. hentzi* clades, were found within given areas. Ten independent runs were carried out in an attempt to test the hypothesis that the ancestral *hentzi* populations were pushed back to or below the defined CRB, the apparent transition and fragmentation region for the *A. hentzi* haplotypes before they spread north during multiple range expansions. Of the 10 runs, all predicted the ancestral population to have occurred in or below the CRB. This hypothesized range of the ancestral population extends from the area around Colorado Bend State Park southwest to the Big Bend National Park area (L =  −365.5) (Figure 1; Table 5). These findings support the hypothesis that the Colorado River Basin was a region of mtDNA haplotype fallback and that central and south Texas was an area of numerous Pleistocene refugia where *A. hentzi* may have persisted before expanding its range northward.

**Table 5:** PhyloMapper predicted ancestral population localities for the *A. hentzi* clade.

PhyloMapper run #	Predicted ancestral location(LAT/LONG)	Likelihood score
Run 1	31.5864N, 98.4727W	−365.50568
Run 2	30.1456N, 101.7626W	−365.5057
Run 3	30.9631N, 101.1786W	−365.50519
Run 4	30.2856N, 100.3240W	−365.50633
Run 5	29.6640N, 101.9184W	−365.50556
Run 6	30.4382N, 100.2284W	−365.50588
Run 7	30.5105N, 101.0496W	−365.50057
Run 8	30.3691N, 100.2488W	−365.49708
Run 9	30.6240N, 100.1777W	−365.50604
Run 10	29.7829N, 102.1015W	−365.50017

## Discussion

We used inferences of phylogenetic relationships and divergence times for a morphologically homogeneous group of spiders to identify the historical events that shaped the evolutionary history of North American tarantulas. With the principal aim to reconstruct the mtDNA lineage history and species boundaries of the *A. hentzi* species group and its sister species in the North American tarantula genus, *Aphonopelma*, we identify potential refugia locations, directionality of range expansion, and test whether the past climate oscillations impacted tarantula diversity and distribution. Our data indicate Pleistocene habitat fragmentation, retraction, and subsequent range expansion events shaped contemporary phylogeographic patterns within *Aphonopelma*, particularly *A. hentzi* post-glacial expansions and dominance of open northern niches.

Intraspecific molecular phylogeographic approaches have been used in the past to identify possible Pleistocene refugia based on the patterns of genetic variation [68], [84]–[92]. The results of the phylogenetic and species delimitation analyses presented here (combined with divergence dating, population expansion rate, and ancestral population reconstruction) show a consistent pattern of past population fragmentation and range expansion that likely occurred during the Pleistocene. New northern niches were opened numerous times during this period allowing *A. hentzi* to dominate and rapidly spread from postglacial refugia in and around Texas' Colorado River Basin. Across the tree, we observe a number of divergence events that occur around this same time period (Figures 3 & 6).

These findings support the hypothesis that events during the Pleistocene shaped *Aphonopelma* diversity and distribution in the southwestern United States. Our use of this phylogeographic approach allows us to interpret the evolutionary relationships of these tarantulas and evaluate how the numerous glacial cycles of the Pleistocene affected the biodiversity of this region, while providing an opportunity to uncover previously unknown “cryptic diversity” in the region.

### Species Crypsis

DNA tree-based methods, in conjunction with the implementation of a “barcode gap” (uncorrected p-distances), and the GMYC species delimitation method consistently recognized a number of divergent and genealogically exclusive groups. These various methods used in concert provide an effective approach to dissecting species boundaries in this spider group; as well they seem to provide strong evidence for a number of previously undiscovered and cryptic species (Figures 3 & 5).

The phylogenetic analyses uncover three major lineages of *Aphonopelma* found in Texas: one closely related group including *A. anax*, *A. armada*, and *A.* sp. *nov 1*, a second closely related group including *A. hentzi*, and *A. moderatum*, and a third group comprising the highly divergent *A.* sp. *nov 2* and *A.* sp. Carlsbad Green (Figures 3 & 4). All putative species clades are separated by >6% genetic pairwise divergence (Table 4). The two species pairs most closely related to each other, according to p-distance, *hentzi-moderatum* and *armada-*sp. *nov 1* are both 6.8% divergent from their sister species. While only slightly higher than the 6% “barcode gap”, the species within these pairs are clearly morphologically identifiable from each other. Such a distinct pattern of reciprocal monophyly is recognized as strong evidence for a lack of gene flow. With genealogical exclusivity, as defined by Velasco [93] where “each member is more closely related to anything else in the group than to anything outside it” consistently seen across the tree, we propose that seven species are delimited in this analysis, two of which are new to science (*A.* sp. *nov 1* and *A.* sp. *nov 2*).

The number of species inferred from the phylogeny and “barcode gap” data corresponds largely with the GMYC analysis. The method, used by a number of authors [26], [61], [94]-[99], correctly delimited *a priori* species groups in five of the seven species previously delimited; *A. anax* and *A. hentzi* comprise lineages designated as multiple clusters (species) by the GMYC method. This reveals strong geographic structuring and phylogenetic gene tree clustering in all putative species groups except *A. hentzi* (Figure 5). Of the seven species, the two cryptic species were found to be sympatric with *A. hentzi* along parts of its southern distribution. Previously recognized as *A. hentzi*, *A.* sp. *nov 1* and *A.* sp. *nov 2* are clearly not conspecifics, as evidenced by high genetic divergence and deep phylogenetic relationships between the three (Figure 3). These three species make up a *hentzi* cryptic species group. The observed case of species non-exclusivity highlights poor taxonomic and identification methods used in the past.

Misidentification of taxa can have serious consequences in the literature affecting our knowledge of nature's patterns and processes [100]. Past ecological and behavioral research was thought to have been carried out on *A. hentzi* [101], *A. echinum* (part of the *hentzi* complex) [102], [103] and *A. steindachneri* (historically misidentified as residing in Texas when it is a California species) [104], but in reality may represent findings for different, highly divergent species (*A.* sp. *nov 1* or *A.* sp. *nov 2*). Studies of several mygalomorph spiders have likewise revealed “cryptic species” based on evidence from extreme intraspecific population structuring [24], [27], [34], [36], [105]. The discovery within this analysis of two “*A. hentzi*-like” spider species comprising genetically divergent lineages was not necessarily surprising, but confirms the notion that cryptic species are a global phenomenon throughout the entire infraorder Mygalomorphae.

The *A. hentzi* lineage was sampled from a previously defined distribution representing one species [13] in an attempt to evaluate the range of morphologic, genetic, and demographic breadth contained within what was previously the *hentzi* species complex. The GMYC clustering clearly separates this lineage into many independently evolving groups despite a lack of any apparent barriers to gene flow among populations. This lack of geographical exclusivity in the *A. hentzi* clustering (6 clusters and 3 singletons) appears to be an artifactual remnant of multiple refugia located in central and south Texas during the Pleistocene. Most haplotype clusters contain individuals from within the defined CRB, with only one comprising individuals solely from south Texas (Del Rio and Comstock), as well as two singletons from SW Texas (Big Bend and Black Gap). Such a pattern could indicate cryptic species within the *A. hentzi* lineage as a consequence of secondary contact or sympatric speciation. However, it is our opinion (based on the data) that these patterns are more likely an artifact of Pleistocene refugial structuring of haplotypes. Likewise, the *A. anax* lineage contains GMYC identified clusters separating the northern and southern populations. This splitting, which appears to be a Pleistocene fragmentation artifact as well, occurs in the northernmost area of the *A. anax* range where “north” and “south” haplotypes overlap in the CRB but are not shared.

The detection of intraspecific GMYC clusters representing previously isolated populations, and therefore erroneously delimited species, has been found in other studies [95]. That being said, the clustering found herein is consistent with the PhyloMapper ancestral locality reconstruction that predicted the ancestral region for the *A. hentzi* clade to have originated southwest of the defined CRB, an area where *A. hentzi* can be found sympatric with southern and western species (Figure 1). It is often thought that populations which become restricted to refugia will often diverge or go extinct through changes in population size due to stochastic events, leading to a lower genetic diversity in the newly colonized areas (as seen north of the Colorado River), lower than in the parental populations remaining in the glacial refugium [106], something we feel is present in our data.

The lack of phylogenetic resolution in the *A. hentzi* clade, combined with short branch lengths and highly divergent southern haplotypes indicate a recent divergence from an ancestral area. The comb-like pattern of a number of the northern *A. hentzi* populations on the tree, linked with the widely distributed closely related haplotypes, population-expansion analyses, and ancestral population reconstruction, support a rapid expansion from refugia within or near the CRB. The large degree of genetic divergence between the southern and northern *A. hentzi* groups suggests that the ancestral populations built up high genetic diversity throughout an area from central to southwestern Texas during Pleistocene fragmentation events.

### The Pleistocene and its effects

Glacial cycles are hypothesized to have transformed a number of North American taxa by shifting species distributions, niches, and population sizes, causing frequent and repeated extinction. As a consequence, large numbers of plants and animals are thought to have experienced a general downward shift in latitude and elevation, occupying a distributional range very different than the one in which they are found today [84], [86]–[88], [92], [107]–[116].

Repeated colonization events during glacial cycles reduced the levels of genetic variation in more recent northern populations, often through population bottlenecks [117], [118]. Hewitt [108], [119] found that if southern populations exhibit considerable haplotype richness in comparison to the lower diversity in northern populations then this southern richness is the result of persistence in refugia and the accumulation of variation over several glacial cycles, while the northern diversity has been caused by rapid postglacial expansion and colonization [119]–[121]. According to Rowe *et al* [117], if ancestral and derived haplotypes do not overlap then ancestral haplotypes should be found close to the origin of range expansion, whereas derived haplotypes are more likely to be found at the leading edge of the range expansion. With a much larger amount of genetic variation maintained south of the CRB and little variation found to the north – i.e. the expanding populations, our findings are consistent with these hypotheses, that the CRB was an *A. hentzi* haplotype fallback zone, a species and genomic transition region, as well as the origin of range expansion.

Potential refugial sites and subsequent range expansions for desert flora and fauna have been predicted to have occurred a number of times in the Chihuahuan Desert region of northern Mexico and southwestern United States [85], [115], [122], [123]. Based on multiple lines of evidence (i.e., paleoclimatic and packrat midden), the Pleistocene climate oscillations in the Chihuahuan Desert (an area of high extant *Aphonopelma* abundance) suggests that desert vegetation (pinyon-juniper woodlands, a shift from the juniper woodlands and juniper grasslands of the upper peripheries of today) was strongly restricted in southern Texas during the wetter and cooler pluvial periods [124], [125] caused by a southward deflection of the jet stream in the presence of the continental ice sheet [126]. If populations were able to persist in northern refugia, north of the Colorado River, it would have been in areas of isolated, protected topography that provided suitable stable microclimates.

In order to test our divergence and biogeographic hypotheses, we employed a combination of the fixed clock rate (4%) previously derived from the mygalomorph genus *Aptostichus* [34], as well as estimating the rate of substitution by calibrating the tree with geologic dates. Our calibrated rate estimation was estimated to be 14.2%, a rate that seems nonsensically high, leading us to predict that splitting events across the tree are older than this rate analysis and that these calibration points are not appropriate for these nodes/groups of specimens.

Divergence dating estimates the fragmentation and subsequent expansion of the “northern” designated *A. hentzi* (*h*CR, *h*CRN, and *h*N) to have occurred around 237,000 ybp (4%) or 8,640 ybp for *h*N (14.2%), 16,230 ybp for *h*CR and *h*CRN (14.2%) (Table 2). Using a calibration date from the Middle Pleistocene limit for the extent of the glaciers (800,000−250,000 ybp), where the glacier ranged south into Kansas stopping in the area east from Topeka to Kansas City [74], [75], seemed a particularly important event for this group of spiders. This event, the most southwestern distribution of the Pleistocene glaciations in North America, likely played a significant role in the distribution and survival of theraphosid spiders in this region. Combined with the circularity of the 14.2% rate analysis, this outcome leads us to conclude that the “true” rate, or most reliable, will be found to reside around this 4% rate of substitution. Based upon this notion, all discussion of results will be in reference to this mygalomorph rate.

The shifting climatic oscillations of the Pleistocene appear to have contributed to the structuring and fragmenting of a number of populations not only within the *A. hentzi* lineage (which possesses an extant range across southern Kansas), but also across the species in this region. Additional cladogenetic events of note support the hypothesis that the Pleistocene played a significant role in the population fragmentation across *Aphonopelma*. The split of all extant *A. hentzi* (*h*) populations was predicted to have occurred around 925,000 ybp, with the *A. hentzi* lineage and the *A. moderatum* lineage splitting at 1.5 million ybp (Table 2; Figure 6). Significant population splits between western and eastern *A. moderatum* lineages, and northern and southern populations of *A. anax*, appear to be timed with the same historical events that affected *A. hentzi* (∼500,000 ybp). During the same relative time period as the *A. hentzi* and *A. moderatum* split, the *A. anax* lineage split from the *A.* sp. *nov 1*/*A. armada* ancestor, and the *A.* sp. Carlsbad Green and *A.* sp. *nov 2* lineages split (∼1.6−1.5 million ybp) (Figures 3 & 6).

Based upon the amount of genetic divergence within *A. hentzi*, it seems plausible that the southern populations were highly fragmented and separated from each other, as well as from the northern populations, for a considerable period of time. Due to the number of haplotypes shared among populations in the CRB and throughout the northern extent of the *hentzi* range, the northern populations were likely to have been fragmented in refugia, periodically coming back into contact thereby reintroducing gene flow during the interglacials. Haplotype sharing most often occurs among the *A. hentzi* populations from within the CRB and populations to the north (Figure 2), indicating that these haplotypes likely dispersed north later in the Pleistocene, dominating the landscape throughout north Texas, Oklahoma, Arkansas, Missouri, Kansas, and SE Colorado. The expansions and retractions of the glaciers during the Pleistocene and an expansion of the prairie biome to the north allowed refugial populations and southern distributions (particularly *A. hentzi*) to expand their ranges and reintroduce gene flow.

### Conclusions

Often referred to as “The Texas Brown tarantula”, this non-descript name belies the truly impressive biology and interesting evolution of this spider lineage. This species' history begins to take shape via multiple lines of evidence -- a story of divergence in allopatric Pleistocene refugia with subsequent range expansion into sympatry. The data presented herein gives the clearest and most accurate formulation to date of the evolutionary history and current genetic composition of this group of North American spiders.

Barriers to gene flow and geographic distance are important factors determining a species' history by restricting interbreeding opportunities. Bond *et al* [34] hypothesize that the biggest contributing factor to speciation in some groups of mygalomorphs may depend more on the restriction of gene flow rather than ecological specialization. This supposition, supported by Peterson *et al* [127], concludes that speciation may be predominantly a vicariance event with ecological differences developing much later; a similar pattern seemingly playing out not only in *A. hentzi*, but potentially the whole of *Aphonopelma* affected by the Pleistocene.

Seldom can we delineate species based solely on molecular data. DNA barcoding has had many proponents and opponents since its origination [18], [19], [21]–[23], [128]–[133]. And while the use of a DNA “barcode” can be employed to help unravel the “species” problem, this approach and the approach of DNA taxonomy, is likely ineffective when used alone. For example, understanding whether GMYC clusters correspond to real “species” is critical to the proposition that DNA barcoding, or any single marker approach, can be effective for use in species identification. As Ahrens *et al* [94] points out, the extent of population sampling is a fundamental element in the GMYC species delimitation approach because the abruptness of the transition point between cladogenesis and coalescence greatly depends on the number of branches near the tip of the tree (defined as the amount of within-species genetic variation that is quantified). We doubt that the GMYC approach correctly splinters *A. hentzi* or *A. anax* into multiple species (Figure 5) but instead represents an artifact of Pleistocene interactions (described above).

Having been previously confirmed as the dominant species found throughout central and north Texas, the tree-based and barcoding approaches substantiate the suspicions of Murray [13] -- that *A. hentzi* is one species that consists of a very large distribution and high ecological plasticity (both relative to other tarantulas in the US), instead of the historically proposed 11 species [4] (Figure 1). Such a conclusion reflects our opinion that while purely molecular based approaches to species delineation appear to work well in this group of spider, these methods are limited and that more synthetic approaches incorporating additional knowledge about life history, population structure, morphology, and spatial distributions are required to make truly informed decisions about what constitutes an evolutionary lineage recognized as a “species”.

The rapid northward spread of *A. hentzi* identifies a trait of particular interest for future research in this group. Multiple co-distributed taxa that are restricted in range being found next to a widespread species with apparent gene flow and haplotype sharing has been found among other mygalomorphs; *Antrodiaetus unicolor* [24], *Aptostichus atomarius* [36], and *Myrmekiaphila comstocki* and *M*. *foliata* [134]. The high amount of apparent gene flow and mtDNA haplotype sharing among some *A. hentzi* populations clearly reveals that while males disperse quite well throughout their localized distribution, female dispersal is considerable -- a phenomenon considered to be relatively rare in mygalomorphs. Is there a dispersal disparity between *A. hentzi* and the other *Aphonopelma* in this region? What behavioral or ecological advantages does *A. hentzi* possess which allow it to disperse quickly, thereby dominating open niches and potentially excluding other species? Are there differential dispersal rates between males and females? While the rate of dispersal and gene flow is unknown, sampling of nuclear loci and microsatellite data will help answer these questions. Deciphering this information will also help us to understand the evolution of theraphosid dispersal, which is poorly known but critical to understanding how species colonize new areas, and how intraspecific cohesion can be maintained across broad distances.

Because tarantulas are long-lived, low vagility ectotherms, and take a number of years to reach maturity, they have a high potential for population subdivision -- making them ideal subjects for evaluating the role that dispersal and isolation play in speciation pattern and process. Moreover, heightening conservation concerns for this group, like in other mygalomorphs [35], due to human development, habitat destruction, and increased pressures as a consequence of the pet trade, highlight the need for accurate taxonomy, phylogenetic relationships, and classification and revisionary studies. An understanding of the evolutionary relationships of North American tarantulas, and their associated ecological factors, may help mitigate future population and species extinction.

## Supporting Information

Table S1 **Specimen and sequence information for haplotypes used in this study.** (Sex designation: F  =  subadult or adult female; M  =  subadult male; MM  =  mature male; juv  =  juvenile)
(DOC)
