# Supplementary material for: Species Delimitation and Phylogeography of Aphonopelma hentzi (Araneae, Mygalomorphae, Theraphosidae): Cryptic Diversity in North American Tarantulas
Source: PLoS One. 2011 Oct 12;6(10):e26207. doi: 10.1371/journal.pone.0026207 (PMC3192178; doi:10.1371/journal.pone.0026207)
Supplement: Table S1 — Specimen and sequence information for haplotypes used in this study. (Sex designation: F = subadult or adult female; M = subadult male; MM = mature male; juv = juvenile) (DOC) [file pone.0026207.s001.doc]

Table S1. Specimen and sequence information for haplotypes used in this study. (Sex designation: F = subadult or adult female; M = subadult male; MM = mature male; juv = juvenile)

| **SPECIES** | **DATABASE # / HAPLOTYPE NAME** | **COLLECTION LOCALITY** | **LAT/LONG** | **SEX** | **GENBANK ACCESSION NUMBERS** | |
| --- | --- | --- | --- | --- | --- | --- |
|  |  |  |  |  | **ND1-16S** | **CO1** |
| *A. armada* | APH0840/AROH1 | O.H. Ivie reservoir, Coleman Co., TX | 31.842967N, 99.5696W | F | JF907123 | JF803370 |
|  | APH0842/ARBS1 | Big Spring, Howard Co., TX | 32.200867N, 101.476883W | F | JF907125 | JF803372 |
|  | APH0843/ARBS2 | Big Spring, Howard Co., TX | 32.200767N, 101.478167W | F | JF907126 | JF803373 |
|  | APH0845/ARSN1 | Snyder, Scurry Co., TX | 32.682133N, 100.925483W | F | JF907129 | JF803376 |
|  | APH0855/AREC1 | Ector Co., TX | 32.076633N, 102.789983W | F | JF907134 | JF803381 |
|  | APH0922ARWH1 | Westhoff, DeWitt Co., TX | 29.136983N, 97.497033W | MM | JF907135 | JF803382 |
|  | APH0950/AROH2 | O.H. Ivie reservoir, Coleman Co., TX | 31.57695N, 99.66065W | M | JF907137 | JF803384 |
| *A. anax* | APH0800/AAEC1 | El Campo, Wharton Co., TX | 29.19405N, 96.2617W | F | JF907056 | JF803303 |
|  | APH0803/AAEC2 | El Campo, Wharton Co., TX | 29.194133N, 96.261367W | F | JF907059 | JF803306 |
|  | APH0804/AALG1 | La Grange, Fayette Co., TX | 29.914417N, 96.866267W | F | JF907060 | JF803307 |
|  | APH0805/AALG2 | La Grange, Fayette Co., TX | 29.914433N, 96.866283W | F | JF907061 | JF803308 |
|  | APH0809/AALG3 | La Grange, Fayette Co., TX | 29.91445N, 96.86638W | F | JF907064 | JF803311 |
|  | APH0810/AALG4 | La Grange, Fayette Co., TX | 29.887683N, 96.8774W | F | JF907067 | JF803314 |
|  | APH0856/AAKG1 | Kingsville, Kleberg Co., TX | 27.4806N, 97.855767W | F | JF907069 | JF803316 |
|  | APH0858/AACA1 | South Padre Island, Cameron Co., TX | 26.076567N, 97.16315W | F | JF907065 | JF803312 |
|  | APH0859/AACU1 | Cuero, DeWitt Co., TX | 29.1221N, 97.410817W | F | JF907072 | JF803319 |
|  | APH0898/AALG5 | La Grange, Fayette Co., TX | 29.91445N, 96.8664W | MM | JF907066 | JF803313 |
|  | APH0924/AAHA1 | Harlingen, Cameron Co., TX | 26.145617N, 97.661717W | MM | JF907071 | JF803318 |
| *A. hentzi* | APH0812/AHAU1 | Austin, Travis Co., TX | 30.354083N, 97.832567W | F | JF907081 | JF803328 |
|  | APH0813/AHAU2 | Austin, Travis Co., TX | 30.299733N, 97.800283W | F | JF907084 | JF803331 |
|  | APH0821/AHJT1 | Jonestown, Travis Co., TX | 30.4721N, 97.945983W | F | JF907104 | JF803351 |
|  | APH0823/AHCB1 | Colorado Bend S.P., San Saba Co., TX | 31.062733N, 98.504117W | F | JF907085 | JF803332 |
|  | APH0825/AHCB2 | Colorado Bend S.P., San Saba Co., TX | 31.122483N, 98.429117W | F | JF907086 | JF803333 |
|  | APH0827/AHBR1 | Brady, McCulloch Co., TX | 31.1235N, 99.384517W | juv | JF907087 | JF803334 |
|  | APH0828/AHBR2 | Brady, McCulloch Co., TX | 31.123517N, 99.384583W | F | JF907088 | JF803335 |
|  | APH0830/AHBR3 | Brady, McCulloch Co., TX | 31.123883N, 99.38335W | F | JF907090 | JF803337 |
|  | APH0831/AHBR4 | Brady, McCulloch Co., TX | 31.123967N, 99.38325W | F | JF907091 | JF803338 |
|  | APH0832/AHOH1 | O.H. Ivie reservoir, Coleman Co., TX | 31.60165N, 99.5915W | F | JF907093 | JF803340 |
|  | APH0833/AHVV1 | Comstock, Val Verde Co., TX | 29.685683N, 101.17145W | F | JF907094 | JF803341 |
|  | APH0834/AHTU1 | Tulsa, Creek Co., OK | 35.99622N, 96.33341W | F | JF907096 | JF803343 |
|  | APH0835/AHKY1 | Kyle, Hays Co., TX | 29.995N, 97.893867W | F | JF907097 | JF803344 |
|  | APH0836/AHGP1 | Grand Prairie, Dallas Co., TX | 32.728783N, 96.990383W | F | JF907092 | JF803339 |
|  | APH0838/AHDA1 | Dallas, Dallas Co., TX | 32.925783N, 96.745817W | F | JF907098 | JF803345 |
|  | APH0863/AHTR1 | Terlingua, Presidio Co., TX | 29.323867N, 103.617117W | F | JF907099 | JF803346 |
|  | APH0868/AHBG1 | Black Gap WMA, Brewster Co., TX | 29.467633N, 102.837333W | F | JF907109 | JF803356 |
|  | APH0896/AHLE1 | Leander, Williamson Co., TX | 30.57295N, 97.850467W | MM | JF907100 | JF803347 |
|  | APH0905/AHWI1 | Cedar Park, Williamson Co., TX | 30.5015N, 97.847917W | MM | JF907101 | JF803348 |
|  | APH0906/AHAU3 | Austin, Travis Co., TX | 30.298533N, 97.793633W | MM | JF907082 | JF803329 |
|  | APH0907/AHAU4 | Austin, Travis Co., TX | 30.298067N, 97.80215W | MM | JF907083 | JF803330 |
|  | APH0910/AHBU1 | Buda, Hays Co., TX | 30.03265N, 97.89275W | MM | JF907106 | JF803353 |
|  | APH0911/AHAU5 | Austin, Travis Co., TX | 30.417283N, 97.750033W | MM | JF907107 | JF803354 |
|  | APH0912/AHWI2 | Round Rock, Williamson Co., TX | 30.517917N, 97.719917W | MM | JF907110 | JF803357 |
|  | APH0913/AHWI3 | Taylor, Williamson Co., TX | 30.560167N, 97.413333W | MM | JF907108 | JF803355 |
|  | APH0914/AHWA1 | Waco, McLennan Co., TX | 31.553483N, 97.203633W | MM | JF907111 | JF803358 |
|  | APH0927/AHVV2 | Del Rio, Val Verde Co., TX | 29.496167N, 101.04455W | F | JF907095 | JF803342 |
|  | APH0933/AHBB1 | Big Bend, Brewster Co., TX | 29.330733N, 103.536367W | MM | JF907122 | JF803369 |
|  | APH0949/AHJT2 | Jonestown, Travis Co., TX | 30.471967N, 97.94595W | F | JF907119 | JF803366 |
|  | APH0976/AHCB3 | Colorado Bend S.P., San Saba Co., TX | 31.1254N, 98.433083W | juv | JF907121 | JF803368 |
| *A. moderatum* | APH0893/AMVV1 | Comstock, Val Verde Co., TX | 29.685067N, 101.171283W | F | JF907163 | JF803410 |
|  | APH0894/AMVV2 | Comstock, Val Verde Co., TX | 29.685483N, 101.171217W | F | JF907161 | JF803408 |
|  | APH0929/AMVV3 | Del Rio, Val Verde Co., TX | 29.56805N, 101.0745W | MM | JF907162 | JF803409 |
| A. sp. Carlsbad Green | APH0850/ACGSE1 | Seminole, Gaines Co., TX | 32.72695N, 102.660533W | F | JF907140 | JF803387 |
|  | APH0851/ACGSE2 | Seminole, Gaines Co., TX | 32.72795N, 102.661683W | F | JF907141 | JF803388 |
|  | APH0852/ACGSE3 | Seminole, Gaines Co., TX | 32.72815N, 102.661333W | F | JF907142 | JF803389 |
|  | APH0853/ACGSE4 | Seminole, Gaines Co., TX | 32.728217N, 102.6619W | F | JF907143 | JF803390 |
|  | APH0854/ACGSE5 | Seminole, Gaines Co., TX | 32.728517N, 102.661583W | F | JF907144 | JF803391 |
|  | APH0888/ACGSE6 | Seminole, Gaines Co., TX | 32.727067N, 102.660983W | F | JF907145 | JF803392 |
|  | APH0940/ACGEC1 | Ector Co., TX | 32.07805N, 102.780783W | F | JF907146 | JF803393 |
| *A.* sp. *nov 1* | APH0862/AN1HI1 | Animas Mtns., Hidalgo Co., NM | 31.945867N, 108.835417W | F | JF907153 | JF803400 |
|  | APH0864/AN1KC1 | Karnes City, Karnes Co., TX | 28.885483N, 97.902683W | F | JF907149 | JF803396 |
|  | APH0867/AN1VV1 | Langtry, Val Verde Co., TX | 29.814633N, 101.563317W | F | JF907154 | JF803401 |
|  | APH0869/AN1CO1 | Chiricahua Mtns., Cochise Co., AZ | 31.7537N, 109.4284W | F | JF907175 | JF803422 |
|  | APH0931/AN1BG1 | Black Gap WMA, Brewster Co., TX | 29.4867N, 102.862733W | MM | JF907155 | JF803402 |
|  | APH0932/AN1BG2 | Black Gap WMA, Brewster Co., TX | 29.5058N, 102.884817W | MM | JF907156 | JF803403 |
|  | APH0937/AN1DA1 | Davis Mtns., Jeff Davis Co., TX | 30.63155N, 104.278117W | MM | JF907160 | JF803407 |
| *A.* sp. *nov 2* | APH0925/AN2VV1 | Del Rio, Val Verde Co., TX | 29.488717N, 100.907633W | MM | JF907164 | JF803411 |
|  | APH0928/AN2VV2 | Del Rio, Val Verde Co., TX | 29.543617N, 101.079183W | MM | JF907165 | JF803412 |
|  | APH0938/AN2SV1 | Sierra Vieja Mtns., Presidio Co., TX | 30.481883N, 104.624033W | MM | JF907166 | JF803413 |
| **OUTGROUP** |  |  |  |  |  |  |
| *A.* sp. Huachuca 2 | APH0880/AHU2 | Huachuca Mtns., Cochise Co., AZ | 31.339267N, 110.328683W | F | JF907171 | JF803418 |
| *A. behlei* | APH0882/ABL | Strawberry, Gila Co., AZ | 34.444717N, 111.453017W | F | JF907172 | JF803419 |
| *A. mojave* | APH0885/AMJ | Joshua Tree N.P., Riverside Co., CA | 33.842133N, 115.488483W | F | JF907173 | JF803420 |
| *A.* sp. Peloncillo | APH0886/APL | Peloncillo Mtns., Hidalgo Co., NM | 31.469267N, 108.73775W | F | JF907174 | JF803421 |
| *A. eutylenum* | APH0895/AEU | San Gabriel Mtns., Los Angeles Co., CA | 34.2175N, 117.8553W | MM | JF907170 | JF803417 |
| *A.* sp. Hualapai | APH0978/AHA1 | Wikieup, Mohave Co., AZ | 34.698283N, 113.629883W | juv | JF907167 | JF803414 |
| *A.* sp. Hualapai | APH0979/AHA2 | Wikieup, Mohave Co., AZ | 34.70215N, 113.622317W | juv | JF907168 | JF803415 |
| *A.* sp. Huachucha 1 | APH0980/AHU1 | Huachuca Mtns., Cochise Co., AZ | 31.3472N, 110.3208W | F | JF907169 | JF803416 |
